# Psychiatry training goes virtual: the experience of the first online edition of the EPA Research Summer School

**DOI:** 10.1192/j.eurpsy.2022.2192

**Published:** 2022-09-01

**Authors:** R. De Filippis, D. Almeida, U. Cikrikcili, L. Di Lodovico, M. Filip, L. Fusar-Poli, A. Gürcan, D. Gurrea Salas, K. Mieze, G. Mijaljica, C. Noël, P. Nwaubani, M. Pantic, B.I. Pérez Longás, A. Pushko, A.A. Román-Jarrín, M. Santos, K. Silagadze, M. Sorokin, C. Tapoi, C. Hanon, N. Hoertel, A. Raballo, N. Sartorius, M. Pinto Da Costa

**Affiliations:** 1 University Magna Graecia of Catanzaro, Department Of Health Sciences, Catanzaro, Italy; 2 Hospital Prof. Doutor Fernando Fonseca, Department Of Psychiatry, Amadora, Portugal; 3 Artvin State Hospital, Psychiatry, Artvin, Turkey; 4 GHU Paris Psychiatrie, Clinique Des Maladies Mentales Et De L’encéphale, Paris, France; 5 Medical University of Lodz, Adult Psychiatry, Lodz, Poland; 6 University of Catania, Department Of Clinical And Experimental Medicine, Psychiatry Unit, Catania, Italy; 7 Başkent University Medical Faculty, Psychiatry, Ankara, Turkey; 8 Psychiatric Services Aargau, Department Of Psychiatry And Psychotherapy, Brugg, Switzerland; 9 Riga Stradins University, Department Of Doctoral Studies, Riga, Latvia; 10 Crisis and Trauma Unit and Refugee Health Clinic, Psychiatry, Göteborg, Sweden; 11 Université Libre de Bruxelles, Child Psychiatry, Brussels, Belgium; 12 Brighton and Sussex Medical School, Clinical Neuroimaging And Neuroscience, Falmer, United Kingdom; 13 University Clinical Centre Kragujevac, Psychiatry, Kragujevac, Serbia; 14 Hospital Lluis Alcanyís, Psychiatry, Valencia, Spain; 15 Ivano-Frankivsk National Medical University, Department Of Psychiatry,narcology And Medical Psychology, Ivano-Frankivsk , Ukraine; 16 University of Navarra, Pamplona, Psychiatry, Navarra, Spain; 17 Hospital Prof. Doutor Fernando Fonseca, Psychiatry, Amadora, Portugal; 18 Tbilisi State Medical University, Psychiatry, Tiblisi, Georgia; 19 V.M.Bekhterev National medical research center for psychiatry and neurology, The Integrative Pharmaco-psychotherapy Of Mental Disorders, Saint-Petersburg , Russian Federation; 20 Prof. Dr. Alexandru Obregia Clinical Psychiatry Hospital, Psychiatry, Bucharest, Romania; 21 Resource Regional Center of Old age Psychiatry, Old Age Psychiatry, Paris, France; 22 Hôpital Corentin-Celton , Psychiatry, Issy-les-Moulineaux, France; 23 University of Perugia, Center For Translational, Phenomenological And Developmental Psychopathology (ctpdp), Perugia, Italy; 24 Association for the Improvement of Mental Health Programmes (AMH), Psychiatry, Geneva, Switzerland; 25 King’s College London, Institute Of Psychiatry, Psychology & Neuroscience, London, United Kingdom

**Keywords:** early career psychiatrists, psychiatry training, Online education, Summer school

## Abstract

**Introduction:**

The European Psychiatric Association (EPA) Summer School allows psychiatric trainees and early career psychiatrists (ECPs) from all over Europe to meet, network, and learn together. After the 2020 edition being cancelled due to COVID-19, the 10th edition in 2021 focused for the first time on research and was conducted remotely.

**Objectives:**

To provide an overview and feedback about the first Virtual EPA Research Summer School as a new way to encourage international networking during COVID-19.

**Methods:**

The School was organized by the EPA Secretary for Education, and 4 Faculty members. It started with a “breaking the ice session” one week before and then a two-days meeting on 23-24 September 2021 using an online video-platform. This was preceded by all the 21 participants (from 18 different countries) recording a short 4-minute video presentation, which was uploaded and shared with other participants and Faculty.

**Results:**

Participants were divided on a voluntary basis into three working groups: 1) “Drug repurposing: overcoming challenges in pharmacoepidemiology” 2) “Psychopathological research in psychiatry”; 3) “How to conduct a cross-sectional survey?”. The Summer School program was composed of plenary sessions with lectures by the Faculty members, discussion sessions, and working groups time. At the end, each group presented a summary of the work done to the rest of the participants.

**Conclusions:**

Although the remote format limits social interactions during the Summer School, overall participants’ high satisfaction and productivity indicate that not only online formats, but also the topic of research might be covered in future editions.

**Disclosure:**

No significant relationships.

